# Molecular architecture of the fruit fly's airway epithelial immune system

**DOI:** 10.1186/1471-2164-9-446

**Published:** 2008-09-29

**Authors:** Christina Wagner, Kerstin Isermann, Heinz Fehrenbach, Thomas Roeder

**Affiliations:** 1 Forschungszentrum Borstel, Dept. Immunology and Cell Biology, Parkallee 1-40, 23845 Borstel, Germany; 2 Christian Albrechts University Kiel, Zoophysiology, Ohlshausenstrasse 40, 24098 Kiel, Germany; 3 Clinical Research Group "Chronic Airway Diseases", Faculty of Medicine, Philipps-University of Marburg, Baldingerstraße, 35033 Marburg, Germany; 4 Forschungszentrum Borstel, Division of Experimental Pneumology, Parkallee 1-40, 23845 Borstel, Germany

## Abstract

**Background:**

Airway epithelial cells not only constitute a physical barrier, but also the first line of defence against airborne pathogens. At the same time, they are constantly exposed to reactive oxygen species. Therefore, airway epithelia cells have to possess a sophisticated innate immune system and a molecular armamentarium to detoxify reactive oxygen species. It has become apparent that deregulation of epithelial innate immunity is a major reason for the development of chronic inflammatory lung diseases. To elucidate the molecular architecture of the innate immune system of airway epithelial cells, we choose the fruit fly *Drosophila melanogaster *as a model, because it has the simplest type of airways, consisting of epithelial cells only. Elucidating the structure of the innate immune system of this "airway epithelial cell culture" might enable us to understand why deregulatory processes in innate immune signalling cascades lead to long lasting inflammatory events.

**Results:**

All airway epithelial cells of the fruit fly are able to launch an immune response. They contain only one functional signal transduction pathway that converges onto NF-κB factors, namely the IMD-pathway, which is homologous to the TNF-α receptor pathway. Although vital parts of the Toll-pathway are missing, dorsal and dif, the NF-κB factors dedicated to this signalling system, are present. Other pathways involved in immune regulation, such as the JNK- and the JAK/STAT-pathway, are completely functional in these cells. In addition, most peptidoglycan recognition proteins, representing the almost complete collection of pattern recognition receptors, are part of the epithelial cells equipment. Potential effector molecules are different antimicrobial peptides and lysozymes, but also transferrin that can inhibit bacterial growth through iron-depletion. Reactive oxygen species can be inactivated through the almost complete armamentarium of enzymatic antioxidants that has the fly to its disposal.

**Conclusion:**

The innate immune system of the fly's airway epithelium has a very peculiar organization. A great variety of pattern recognition receptors as well as of potential effector molecules are conspicuous, whereas signalling presumably occurs through a single NF-κB activating pathway. This architecture will allow reacting if confronted with different bacterial or fungal elicitors by activation of a multitude of effectors.

## Background

Most animals possess an oxygen delivery system to fulfil the demands of their metabolically active organs. The architecture of respiratory organs is surprisingly similar throughout the animal kingdom, with branched tubules as repetitively used entities. Our own lung is made of a complex network of branching tubes that terminate in alveoli, where oxygen diffuses into the blood. In *Drosophila *larvae, the tracheal system consists of approximately 10.000 interconnected tubes. These very simple tubes are built from an epithelial monolayer that wraps around the central, gas-transporting lumen [1]. Oxygen enters through two pairs of spiracular openings and passes through primary, secondary and terminal branches, reaching all tissues in the body. Although of much simpler organization, the fly's airway system shows striking similarities with our own lung regarding its architecture but also its physiology [2,3]. The simplicity of its organization has made the *Drosophila *airway system to the most informative model for studying the genesis of tubular organs such as the lung or the kidney, and at the same time for complex processes such as angiogenesis [1,4,5].

One major characteristic of most, if not all epithelia, is the ability to launch an immune response if confronted with pathogens such as bacteria, fungi or viruses. This cell-autonomous response, where all parts of the innate immune system, comprising pattern recognition, signal transduction and effectuation, reside in the epithelial cells themselves. Even men depend on this evolutionary most ancient immune system in the fight against infections [6-8]. In addition, defects in the innate immune system of the epithelial cells may be one of the major causes underlying inflammatory diseases of barrier epithelia such as Crohn's disease or chronic asthma [9,10]. A detailed analysis of the inventory of immune-competent epithelial cells has always been obstructed by the complexity of the epithelia of interest. Usually, a number of different cells constitute the epithelia. In addition, infection and a primary immune response of the epithelial cells recruit the entire armamentarium of leucocytes to the site of infection. Amongst all immune competent epithelial organs, the insect airway epithelium is presumably the simplest one. It comprises only one type of epithelial cells, organized in an epithelial monolayer, thus representing a "cell culture" in the intact animal [4,11].

*Drosophila *has served over decades as a tremendously useful model to study basic mechanisms in almost every area of modern biomedical research. This holds also true for innate immunity that has experienced a revival following pioneering work in *Drosophila *[12,13]. Numerous studies performed in this field gave us a comprehensive picture of the fly's immune response towards invading microorganisms. In contrast, our knowledge about the epithelial immunity is only fragmentary so far. We know that various epithelia respond to pathogen encounter with the expression of antimicrobial peptide genes [14]. In addition, the IMD-, but not the Toll-pathway is of central importance for this reaction [15].

To improve the prospects of this surprisingly simple model epithelium that has been and will be used in numerous research areas, we performed an extensive transcriptome study with the aim to better understand different lung diseases such as asthma, COPD or acute lung injury [16]. Therefore, we have focuses on three areas, innate immunity, response to reactive oxygen species and signalling.

## Results

**Figure 1:**
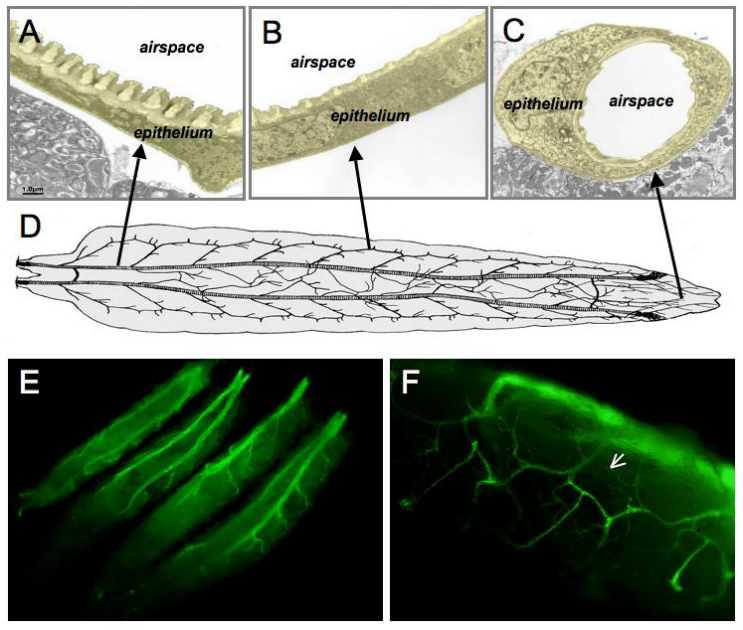
**Organization of the fruit fly's airway epithelium**. The airway system of the Drosophila larvae is made of simple tubes in a hierarchical order (D, modified after [31]). In all airways, starting from the primary (A) over secondary (B) up to terminal branches (C), a single layer of epithelial cells wraps around the central air-filled tube. If confronted with bacteria (*Erwinia carotovora *or *Pseudomonas aeruginosa*), the airway epithelium reacts with the expression of antimicrobial peptides (visualized using a drosomycin::gfp reporter, E). All cells, even the most terminal structures, are able to mount an immune response (arrow, F).

The airways of the fruit fly's larva show a very simple organization. A hierarchic organization of this oxygen transport system of tubes is made of primary, secondary and terminal branches. In all these regions, this organ is built of only a single layer of epithelial cells covered by a cuticular intima (Fig 1A–D). If confronted with bacteria (in this case *Erwinia carotovora*), this epithelium is able to launch an immune response, visualized by the expression of the *gfp *gene, which is under transcriptional control of the *drosomycin *promoter, an important antimicrobial peptide of the fly (Fig. 1E). The hypothesis that all airway epithelial cells are homogenous in terms of their immune reaction is supported by the observation that all these cells, even those that build the finest tracheal endings on the target organs, show an activation of the *drosomycin *gene transcription following an infection (Fig. 1F).

### Immuno-transcriptome of the airway epithelium of the fruit fly

To uncover the architecture of the tracheal epithelial cell's immune system, we looked at the presence of all known constituents of the fruit fly's innate immune system. Manual isolation of trachea from third instar larvae was performed prior to RNA isolation. The material was thoroughly purified from attached, non-tracheal material. It was checked for contamination with fat body or hemocyte material by RT-PCR with primers derived from genes exclusively expressed in either of these tissues (*P6 *and *hemese *respectively). Only if these controls revealed negative results, the material was used for downstream experiments. We looked at the pattern recognition receptors, the molecules that constitute different signalling pathways involved in innate immune responses as well as at relevant transcription factors.

### Pattern recognition receptors

**Figure 2:**
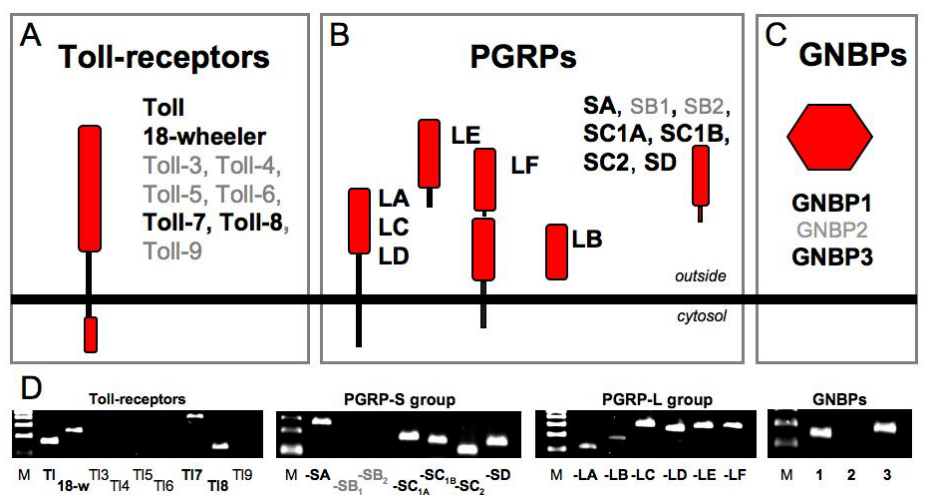
**Pattern recognition receptors of the airway immune system**. To identify the genes coding for pattern recognition receptors in airway epithelial cells, we performed RT-PCR experiments with RNA derived from thoroughly isolated epithelial cells. Regarding the Toll-receptors, only 4 out of 9 are expressed in these cells (A, D). All except 2 peptidoglycan recognition proteins (PGRPs) are present in the airways (B, D). From the gram-negative binding proteins, GNBP1 and 3 are present (C, D). Positive controls were performed with fatbody and blood cell derived RNA, negative control without template.

Three different groups of pattern recognition receptors are known to be relevant for the fly's immune response, namely the Toll-receptors, the Gram-negative binding proteins (GNBPs) and, most importantly, the peptidoglycan recognition receptors (PGRPs). We tested for the expression of all members of these families. Among the 9 Toll receptor genes of the fruitfully, four, namely *toll *itself, *18 wheeler*, *toll-7 *and *toll-8 *are expressed in the airway epithelium (Fig. 2A, D). Two out of three GNBPs, GNBP 1 and 3 are also present in the tracheal tissue (2C, D). Most notably, a variety of different members of the PGRP gene family are expressed in this organ. The members of the family can be subdivided into two major groups, small PGRPs that are soluble and large ones, where some members are thought to be membrane bound. Among the small representatives, only PGRP SB1 and SB2 are not expressed in the airway epithelium. In addition, all members of the large family of PGRPs, namely PGRP LA, LB, LC, LD, LE, and LF, are present in the tracheal epithelium (Fig. 2B, D).

### Signaling pathways

**Figure 3:**
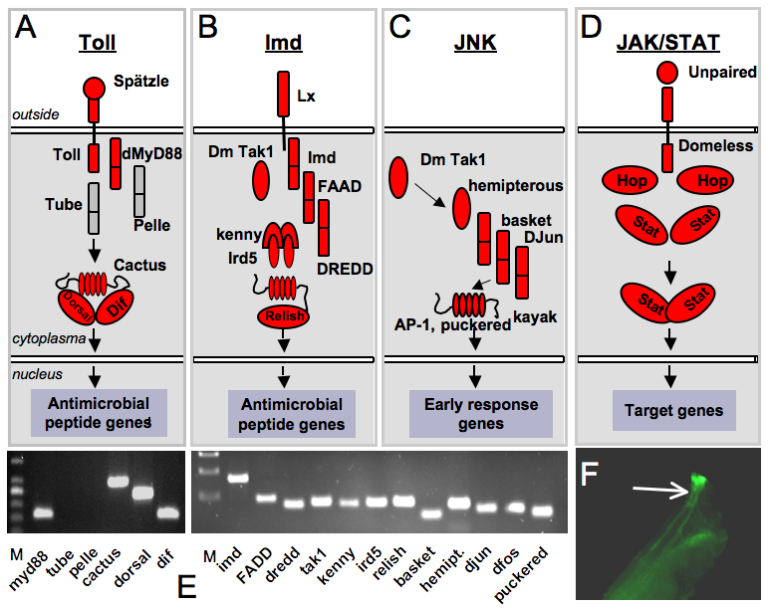
**Signal transduction pathways in the airway epithelia**. Among the 4 signal transduction pathways relevant for the innate immune response of the fly, only the Toll-pathway is not represented by all vital members (A, E). In opposite, the other pathway that terminates into activation of NF-kB factors, the IMD-pathway, is functional, because all corresponding genes are expressed (B, E). The JNK-pathway should also be functional because all relevant members are present (C). JAK/STAT signalling depends only on a very limited number of genes. The complete set of genes required for its activation is expressed in the airways (D). In addition, JAK/STAT-signalling can be visualized in the terminal tracheal structures using a STAT::gfp reporter system (F, 19).

Four major signaling pathways are believed to mediate the effects in *Drosophila *innate immunity. These are the Toll-, the IMD-, the JAK/STAT-, and the JNK-pathways. We looked for expression of all relevant constituents of these four major signaling pathways in the airway epithelium by RT-PCR. Regarding the Toll-pathway, we found that only a fraction of members of this pathway is present in the airway epithelium. Among the genes expressed are the cytokine *spätzle*, *Toll *itself, its adapter protein *MyD88 *and the NF-κB homologues *dorsal *and *dif*, but also their repressor *cactus*. Other essential parts of this pathway (*tube *and *pelle*) are obviously not expressed in the tracheal epithelial cells (Fig. 3A, E).

Regarding the second, major signaling pathway, the IMD-pathway, a different scenario emerged. All members required for proper function of this pathway are expressed in the airway epithelia. Starting with the mentioned above PGRPs, IMD itself, TAK1, FADD, Dredd, Kenny, Ird5 and relish are present. This confirms that the IMD-pathway is functional in the airway epithelium meaning that bacterial patterns can be recognized and this information transformed into a suited physiological response (Fig. 3B, E).

The JNK pathway is relevant for the control of immune responses in the fly. Its exact role for the activation of a proper immune response is still matter of debate. Apparently, it is a discrete pathway [17] that may have an inhibitory effect on the IMD pathway [18]. Key components of this pathway including Tak1, hemipterous, basket, d-Jun and dFos, are expressed in the airway epithelium, indicating that this pathway is also functional (Fig. 3C, E). The fourth pathway associated with immune responses, the JAK/STAT pathway, consists of only a very limited number of elements. The ligand *upd *(one of the 3 cytokines upd, upd2 and upd3) binds to the receptor *domeless*, which activates the Janus kinase hopscotch (hop) and finally the STAT transcription factor. All members of this pathway are present in the larval airway epithelia (Fig. 3D). These results are summarized schematically in Figure 3. In addition, STAT-dependent transcription can be visualized using transgenic flies, where *gfp*-expression is under the control of STAT-responsive elements [19]. Larvae of these flies show a pronounced *gfp*-expression in the tracheal endings, the spiracles up to early L3 stages (Fig. 3F).

### Transcription factors of the immune system

**Figure 4:**
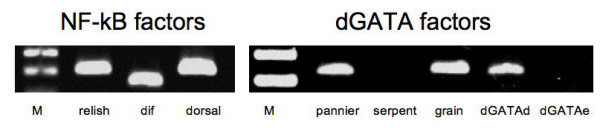
**Transcription factors relevant for the immune response**. RT-PCR analysis of transcription factors relevant for immune responses. All 3 NF-kB factors are present in the airway epithelium. In addition, 3 out of 5 GATA-factors were found in these cells.

Transcription factors are of central importance for the execution of immune related signaling pathways. In addition to the entire set of Drosophila NF-κB factors (relish, dif and dorsal), three out of five GATA factors, namely pannier, grain and dGATA-d, are also present in the airway epithelial cells. In contrast to them, serpent and dGATAe could not be detected (Fig. 4).

### All three functional signaling pathways are part of the airways epithelial immune system

**Figure 5:**
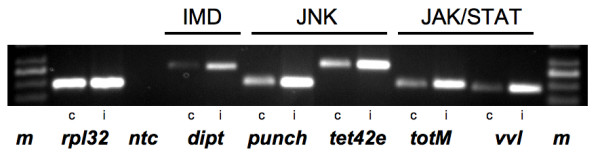
**Infection with *P. aeruginosa *provokes increased expression of target genes of the imd-, JNK- and JAK/STAT-pathways**. Semiquantitative RT-PCR analysis of target genes for the corresponding immune relevant pathway was performed with control material (c) and material from infected larvae (i). Comparison with the house keeping control gene rpl32 reveals that all candidate genes (dipt = diptericin, punch, tet42e = tetraspanin 42e, totM = turandot M vvl = ventral vein lacking) are expressed at higher levels in infected trachea.

Material derived from larval trachea infected with *Pseudomonas aeruginosa *was used to evaluate if the three signaling pathways the IMD-, the JNK-, and the JAK/STAT-pathway are functional in the airway epithelium and that they participate in orchestrating the immune response. Diptericin (dipt) as a typical IMD-pathway gene, punch and tetraspanin 42e (tet42e) as typical JNK-pathway genes and TurandotM (totM) and ventral veins lacking (vvl) as typical JAK/STAT-induced genes were tested with this material. Semiquantitative RT-PCR with equal amounts of cDNA (infected trachea vs. control meaning non-infected material) showed that expression of these representative genes was increased whereas expression of the housekeeping gene rpl 32 was not (Fig. 5).

### Transcriptome of the airway epithelium

**Figure 6:**
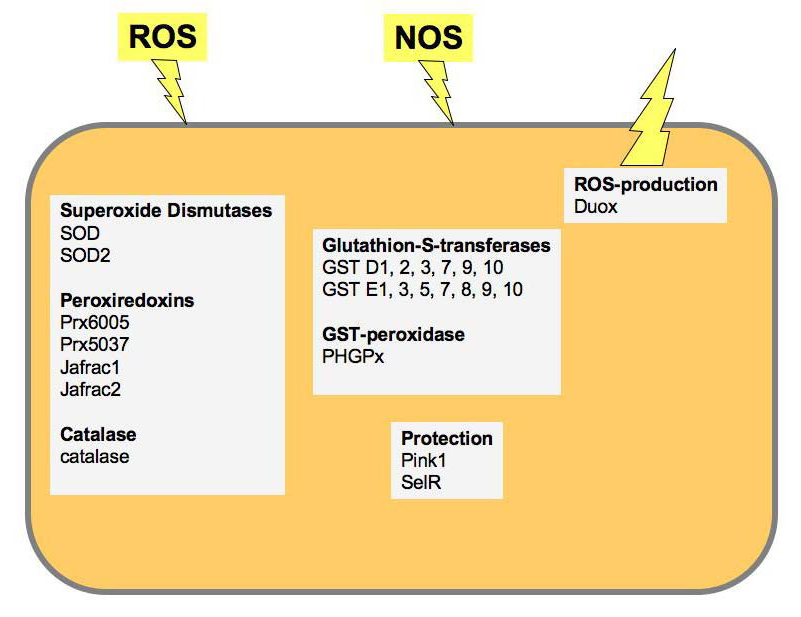
**Schematic delineation of antioxidant enzymes in the airway epithelial cell**. A number of different enzymes that have the ability to detoxify reactive oxygen as well as reactive nitrogen species (ROS, NOS) are present in airway epithelial cells. Especially the complete SOD family and a total of four peroxiredoxins might serve as the first line of defence against oxygen radicals. On the other hand, a sophisticated glutathione based system allows detoxification of various different compounds. The dual oxidase Duox may be used to produce ROS in response to an encounter with bacterial pathogens.

The identification of genes that are expressed and of those that are preferentially expressed in the airway epithelium was achieved using DNA-microarray studies. Tracheal tissues were isolated from early third instar larvae and purified from contaminating tissues. In addition, we excised trachea from larvae of the same age and used the remaining material (whole larval animals without their major trachea) as control. This experimental setup gave us two different types of results, 1) the airway epithelial transcriptome and 2) the genes that are preferentially expressed in these cells. Using a relatively stringent set of criteria (at least 75 pixel above background in all experiments), we identified a list of 3100 genes. This is definitely not the complete set of genes that are expressed in these cells, especially because low abundant transcripts might have escaped our criteria, but we can be relatively sure that the corresponding transcripts are present [see Additional file 1]. Here, we focused on three aspects only: 1) immune effectors, 2) response to oxidative stress, and 3) signaling through G-protein coupled receptors. 1) In non-infected animals, some antimicrobial peptide genes show a basal level of expression. These are defensin, metchnikowin, drosomycin, attacin A and diptericin. In addition, 3 out of 8 lysozymes, namely LysS, LysB and LysX, are present in these cells. 2) Regarding the capacity to cope with different oxidative stressors, the airway epithelium has a tremendously diverse armament of antioxidant enzymes. Detoxification of reactive oxygen species can be done with either of the two superoxide dismutases (SOD1 and SOD2), 4 out of 5 peroxiredoxins (two of them allowing thioredoxine as acceptor) and an impressive number of glutathione-S-transferases (GST; Fig. 6). Hydrogen peroxide can be converted to water through catalase activity. In addition, Duox, a dual oxidase is present, an enzyme known to produce reactive oxygen species in response to an infection in the gut [20]. Signaling through G-protein coupled receptors is the most important way to adapt cells to different physiological situations. Only a very limited number of G-protein coupled receptors are present in higher amounts in the airway epithelial cells, namely a methusaleh receptor (meth-l3), two neuropeptide receptors (NPFR1 and Takr86C) as well as an octopamine receptor (octß-2R). Whereas we have no information regarding the relevance of the first three receptors, it is known from studies using larger insects, that the biogenic monoamine octopamine modulates the activity of airway epithelium, presumably mediated via the octß-2R receptor [21].

The set of genes that show a predominant expression in the tracheal epithelium was identified by comparing the expression data from the airway epithelium with those of the remainders of the animals. All genes showing an at least 2 fold higher expression in the trachea compared with the remainder of the larvae were chosen, yielding a total list of 413 genes [see Additional file 2]. We selected 10 out of them randomly and used quantitative RT-PCR to verify the tracheal-specific expression, which was successful (data not shown). Three of them, namely CG6074, CG13640 and CG18105, showed a very high degree of specificity with negligible expression in the remainder of the larvae. A small number of the genes with trachea specific expression, scilicet the top 50 annotated genes are summarized in Table 1. Remarkably, *transferrin 1 *is among the genes showing highest specificity for the airway epithelium, pointing to a vital role of this iron catching molecule for the airway epithelial physiology. High concentrations of transferrin will result in an iron-depletion of the epithelial surface environment, therewith inhibiting growth of most bacteria [22].

**Table 1:** Genes specifically expressed in the airway epithelium.

	**Gene-ID**	**Gene-symbol**	**Gene-name**	**Ø log2 ratio**	**covariance**
**1**	CG6186	Tsf1	Transferrin1	4.1	0.05
**2**	CG18105	ETH	Ecdysis triggering hormone	3.61	0.06
**3**	CG2520	Lap	like-AP180	3.32	0.08
**4**	CG10279	Rm62	Rm62	2.99	0.27
**5**	CG2139	aralar1	aralar1	2.37	0.03
**6**	CG9020	Aats-arg	Arginyl-tRNA synthetase	2.32	0.18
**7**	CG1780	Idgf4	Imaginal disc growth factor 4	2.28	0.09
**8**	CG7539	Edg91	Ecdysone-dependent gene 91	2.26	0.23
**9**	CG3284	RpII15	RNA polymerase II 15 kD subunit	2.21	0.19
**10**	CG18076	shot	short stop	2.16	0.27
**11**	CG14887	Dhfr	Dihydrofolate reductase	2.1	0.35
**12**	CG9334	Spn3	Serine protease inhibitor 3	2.1	0.13
**13**	CG1743	Gs2	Glutamine synthetase 2	2.09	0.24
**14**	CG8409	Su(var)205	Suppressor of variegation 205	2.06	0.26
**15**	CG13098	mRpL51	mitochondrial ribosomal protein L51	1.98	0.24
**16**	CG6302	l(3)01239	lethal (3) 01239	1.96	0.06
**17**	CG13628	Rpb10	Rpb10	1.9	0.09
**18**	CG10944	RpS6	Ribosomal protein S6	1.89	0.28
**19**	CG3054	l(2)k05819	lethal (2) k05819	1.81	0.25
**20**	CG4337	mtSSB	mitochondrial single stranded DNA-binding protein	1.8	0.11
**21**	CG5258	NHP2	NHP2	1.8	0.06
**22**	CG30498	boca	boca	1.78	0.07
**23**	CG4665	Dhpr	Dihydropteridine reductase	1.78	0.29
**24**	CG11797	Obp56a	Odorant-binding protein 56a	1.75	0.17
**25**	CG16792	DebB	Developmental embryonic B	1.73	0.12
**26**	CG3949	hoip	hoi-polloi	1.72	0.19
**27**	CG4457	Srp19	Signal recognition particle protein 19	1.72	0.2
**28**	CG5170	Dp1	Dodeca-satellite-binding protein 1	1.66	0.07
**29**	CG4464	RpS19a	Ribosomal protein S19a	1.64	0.07
**30**	CG11921	fd96Ca	forkhead domain 96Ca	1.63	0.24
**31**	CG9670	Fal	falten	1.63	0.15
**32**	CG10679	Nedd8	Nedd8	1.62	0.21
**33**	CG8604	Amph	Amphiphysin	1.61	0.06
**34**	CG10624	sinu	sinuous	1.6	0.17
**35**	CG11271	RpS12	Ribosomal protein S12	1.59	0.45
**36**	CG10603	mRpL13	mitochondrial ribosomal protein L13	1.57	0.38
**37**	CG12665	Obp8a	Odorant-binding protein 8a	1.57	0.46
**38**	CG4584	dUTPase	Deoxyuridine triphosphatase	1.57	0.29
**39**	CG10596	Msr-110	Msr-110	1.55	0.12
**40**	CG11979	Rpb5	Rpb5	1.53	0.39
**41**	CG11482	Mlh1	Mlh1	1.52	0.1
**42**	CG3379	His4r	Histone H4 replacement	1.51	0.12
**43**	CG16869	Ance-2	Ance-2	1.5	0.23
**44**	CG4494	smt3	smt3	1.45	0.17
**45**	CG3035	Cm	carmine	1.44	0.07
**46**	CG7977	RpL23A	Ribosomal protein L23A	1.44	0.47
**47**	CG3751	RpS24	Ribosomal protein S24	1.43	0.15
**48**	CG3595	sqh	spaghetti squash	1.42	0.11
**49**	CG3450	l(2)k03203	lethal (2) k03203	1.41	0.32
**50**	CG32854	mRpS21	mitochondrial ribosomal protein S21	1.4	0.41

List of genes with a significantly higher level of expression in the tracheal tissue compared with larval tissues minus trachea. The 50 annotated genes with highest specificity for tracheal expression are listed.

**Figure 7:**
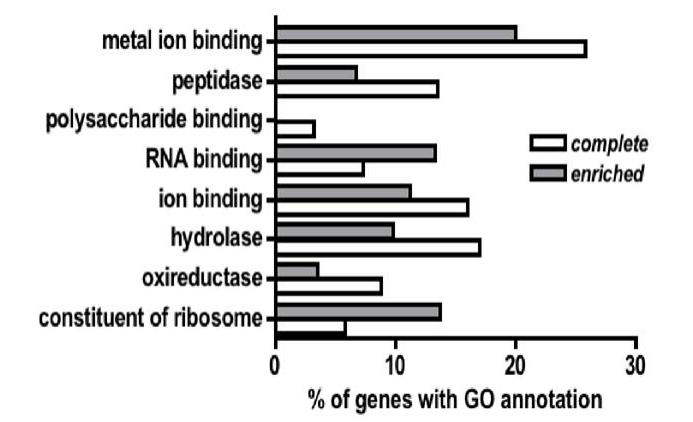
**Occurrence of functional annotation groups in the complete airway epithelial transcriptome and within the group of genes with preferential expression in the airway epithelium**. Functional annotation of both sets of genes was performed using the Fatigo+ program package (23). Exemplary, some GO annotations analysis of the molecular function level 3 (structural constituent of ribosome, oxidoreductase, hydrolase, ion binding) and 4 (RNA binding, polysaccharide binding, peptidase, metal ion binding) are listed. All groups show a statistically significant (p < 0.05) difference in their occurrences between the two sets of genes.

A comparison between the two sets of genes (complete transcriptome versus genes enriched in airway epithelial cells), using the Fatigo+-tool [23] revealed some interesting aspects. Gene ontology analysis of the molecular function of annotated genes revealed a surprisingly high number of ion binding (level 3) and especially metal ion binding proteins (level 4). Differences between the two sets of genes are especially seen for structural constituents of ribosomes and RNA binding (higher in the airway epithelia enriched fraction) as well as oxidoreductase, hydrolase, peptidase and polysaccharide binding (higher in the complete transcriptome, Fig. 7).

## Discussion

Airway epithelia are characterized by common architectures throughout the animal kingdom. Efficient gas exchange requires maximized surface areas and minimized epithelial thickness. These features are directly opposed to the needs of an immune response that favors minimization of surface areas and robust design of the epithelia. This conflict of interest has to be attenuated by very effective immune responses inhibiting bacterial colonization and growth rapidly and effectively.

The repertoire of these epithelial cells with immune related proteins defines their potential defense response. Infection with different bacteria can obviously induce a pronounced immune response in the airway epithelia of the fly. Apparently, this reaction relies on the IMD-pathway, a feature that is presumably common to all epithelial tissues [14,15]. The molecular rationale behind this focus on the IMD-pathway might be very simple; all vital members of the IMD-pathway are present in the airway epithelial cells. This allows a cell-autonomous activation of this pathway, finally leading to expression of antimicrobial peptide genes. In contrast, the other immune-relevant pathway leading to activation of NF-κB factors, the Toll-pathway is not complete in the airway epithelial cells. Some vital members of this pathway are simply not present in these cells, obviously obstructing activation of the entire pathway. Present are the receptor Toll, the ligand spätzle, the adaptor MyD88 and the complex of both NF-κB factors dorsal and dif as well as their repressor cactus. Especially the presence of the entire NF-κB complex may ensure that dorsal or dif are not activated. Setting the Toll-pathway aside in epithelial immunity might be a reasonable if not an essential strategy. Epithelial responses are first and foremost local responses to prevent the epithelium from unwanted immune reactions. The Toll-pathway is on principle an organ systemic signaling system, because the recognition steps occur within the extracellular space and if the recognition cascade is activated, all responsive cells having contact with this extracellular space are activated. In case of the airway epithelium this would mean a reaction of all airway epithelial cells if the Toll-pathway is activated locally in this structure. Expression of the drosomycin gene, which is known to be a classical Toll-pathway dependent gene, is hard to understand, but this seemingly paradoxical situation has been reported earlier [14]. Regarding the pattern recognition receptors, a great variety of PGRPs (peptidoglycan recognition receptors) and GNBPs (gram negative binding proteins) are present in this tissue. Especially all membrane bound PGRPs are present, presumably allowing sensing a great variety of different pathogen associated molecular patterns (PAMPs). Although this tissue expresses this wealth of pattern recognition receptors, the response should be relatively stereotype, simply because all these receptors converge onto a single signal transduction pathway, namely the IMD-pathway. Nevertheless, two other immune-relevant pathways that reside in the epithelial cells, the JNK- and the JAK/STAT-pathways, may shape the response towards an encounter with pathogens. Both pathways are present in these cells, suggesting that they are functional. Regarding the terminal parts of the signal transduction pathways, we observed an unexpected complexity. All three NF-κB factors, relish, dorsal and dif are present in this tissue. This is insofar puzzling as it is generally agreed that dorsal and dif are devoted to the Toll-pathway, which is not functional in the airways. In addition, 3 members of the GATA-family of transcription factors are also present in the airways, presumably playing an important role in the control of immunity, as it has been shown for other tissues [24]. The basal expression of a number of antimicrobial peptide genes as well as of lysozymes indicates that this armament against microbes can be used in the airway epithelium. Another, complementary part of the immune response may be seen in the constitutively high expression of the transferrin1 gene. It is at position 2 of the genes with highest specificity for the airway epithelium (table 1). In the mammalian airway epithelium, transferrin is known to play an important role not only in the capture of Fe^2+^-ions, but even more importantly, it deprives the airway liquid from Fe^2+^, thus inhibiting bacterial growth [25].

Very impressive is the unforeseen complexity of antioxidative enzymes serving the airway epithelial cells. As this structure is directly exposed to high oxygen pressure and environmentally produced reactive oxygen species (ROS), it simply might be imperative using the almost complete antioxidative armament to protect this very delicate structure. Alternatively, ROS production by e.g. the DUOX may be a strategy to fight against pathogenic bacteria entering the airways, thus urging to protect the own cells against these endogenous ROS production. ROS species that might be generated by diverse sources such as pollen are believed to represent major mediators of inflammatory responses in the airway epithelium [26]. Impairments of central antioxidative enzymes such as SODs are therefore of central importance for the development of long lasting airway inflammatory responses [27].

Signaling in the airway epithelium is not yet understood at all. It is known that adrenergic signaling has an important impact on the development of asthma, with the epithelial cell being in a central position. Nevertheless, we have no idea, what is regulated in the airway epithelial cells in response to this stimulus [28]. In insects, octopamine, the invertebrate adrenaline, increases cAMP in the trachea [21], similar as in the vertebrate system, but so far, the physiological relevance of this hormonal modulation in not understood.

## Conclusion

Airway epithelial cells have to cope with a multitude of problems. They come into contact with an unpredictable diversity of airborne bacterial and fungal spores. Presumably to deal with this problem, the innate immune system of the fly's airway epithelial cell has a very peculiar architecture. The almost complete set of pattern recognition receptors, especially the membrane bound ones, should enable to detect the vast majority of these airborne pathogens. They converge onto only one NF-κB activating signalling cascade, the IMD-Pathway. Omitting the second signalling cascade that converges onto NF-κB factors, the Toll-pathway, may be a necessity of epithelial immune systems to restrict the response locally. Shaping of the immune response may occur through additional signalling systems such as the JNK- and the JAK/STAT-pathway. Epithelial cells obviously contain the almost complete set of enzymatic antioxidants, including both SODs, 4 out of 5 peroxiredoxins, the catalase and various glutathione-S-transferases, presumably to cope with exogenously generated reactive oxygen species. The great potency of airway epithelial cells to fight pathogens and to cope with reactive oxygen species and the willingness to launch the corresponding responses may represent a major reason why these structures are prone to various inflammatory diseases such as asthma or COPD (chronic obstructive pulmonary disease).

## Methods

### Molecular biology

Trachea of early third instar larvae were prepared manually in ice-cold PBS. Subsequently, purified trachea were transferred to the denaturation solution of the RNA isolation kit and immediately homogenized. RNA isolation was performed with the RNA NucleoSpin kit (Macherey-Nagel, Dueren, Germany). CapFinder cDNA-synthesis was performed as described earlier [29]. Amplification of the cDNA was performed for 28 cycles taking advantage of a long and accurate PCR system. The integrity and quality of the amplificate was checked by gel electrophoresis. This material was used for RT-PCR experiments, qRT-PCR experiments and the production of labeled hybridization probes for DNA-microarray analysis. cDNA was used for downstream applications only if RT-PCR with primers for *hemese *(hemocytes) and *P6 *(fat body) didn't gave any signal. RT-PCR was performed with corresponding primer pairs for every gene under investigation (see supplementary information). The amplification was performed for 30 cycles using a conventional PCR-approach. Positive (fat body and hemocytes as template) as well as negative controls (no cDNA-synthesis) were always included. Semiquantitative RT-PCR was performed with trachea isolated from control animals and those infected with *Pseudomonas aeruginosa*. Infection was essentially performed as described [14]. RT-PCR was performed for 30 cycles with identical amounts of cDNA using the house keeping gene rpl 32 as control. Other infection experiments were perfomed with the insect pathogen *Erwinia carotovora *as described [14]. Quantitative RT-PCR was performed with a Lightcycler (Roche Diagnostics, Ingelheim, Germany) using the kit TAQurate™ Green Real-time PCR master mix (Epicentre Technologies, Biozym, Hess. Oldendorf, Germany). Probe sets were normalized against the housekeeping gene rpl 32. At least three independent experiments were used.

For microarray analysis, equal amounts of amplified and purified cDNA were subjected to T7-based cRNA synthesis. The synthesis was performed with the T7 MEGAscript kit (Ambion, Applera, Darmstadt, Germany) and supplemented aaUTPs (Ambion) for the labeling of the cRNA. Following purification (RNA NucleoSpin kit, Macherey-Nagel, Düren, Germany) and subsequent precipitation of the cRNA, approximately 10 μg of aminoallyl modified-cRNA was coupled to succinimidyl modified-Cy3 und -Cy5 dyes (Amersham) in the presence of 50% DMSO and 0.05 M NaHCO_3 _(pH:9.0). Coupling reaction was carried out for two hours in the dark followed by purification and precipitation of the labeled cRNA. After assessment of the labeled cRNA approximately 2–3 μg (or 150 pmol) of Cy3 and Cy5 labeled probe were used for hybridization. Hybridisation was carried out at 42°C overnight. After hybridization slides were washed twice with 1 × SSC, 0.1% Triton-X-100 at 60°C for 15 minutes and with 0.1 × SSC, 0.1% Triton-X-100 at 37°C for 15 min. Subsequently they were washed with 0.1 × SSC for 30 seconds at room temperature and rinsed with water before dried.

### Microarray analysis

Gene expression analysis was performed by using the Drosophila OLIGO 14k_version1 gene chip (Canadian Drosophila Microarray Center, University of Toronto, Canada). The slides were scanned by using the GenePix™ 4000B scanner (Axon Instruments, Molecular Devices, München, Germany). For spot finding and generating preliminary result files the raw scanned image files were analyzed using GenePixPro version 6.0 whereas data normalization, quality assurance and control, filtering and clustering were carried out with GeneTraffic (Iobion, Agilent, Waldbronn, Germany) and statistical analysis with the SAM-program package. In search of functional composition of genes significantly affected upon infection and ectopic expression we used the bioinformatics web tool FatiGO [23].

The DNA-microarray experiments have been deposited in the GEO-database.

### Electron microscopy

*Drosophila *larvae were fixed simultaneously with 1.5% glutaraldehyde and 2% osmium tetroxide in 0.1 M sodium cacodylate buffer for 90 minutes on ice [30]. After rinsing with 0.1 M sodium cacodylate buffer, samples were post-fixed with 1% osmium tetroxide in 0.1 M sodium cacodylate buffer for 2 hours, rinsed in the same buffer (4 × 5 min), washed in distilled water (2 × 5 min), and stained en bloc in half-saturated uranyl acetate over night. After rinsing with distilled water (4 × 5 min), samples were dehydrated through an ascending series of actone (70%, 90%, 100% two times for 10 min each), transfered into a 1:1-mixture of acetone and Araldite for one hour, and finally into pure Araldite over night. After transfer into fresh resin, samples were polymerised at +60°C for three days. Ultrathin sections were cut on an Ultracut E (Reichart-Jung, Wien, Austria), collected on formvar coated nickel grids, stained with lead citrate, and analysed using a Zeiss EM 900 (Zeiss, Oberkochen, Germany).

## Authors' contributions

CW and KI carried out the molecular genetic studies. HF carried out the electron miscroscopic studies. TR conceived the study, and participated in its design and coordination and drafted the manuscript. All authors read and approved the final manuscript.

## Supplementary Material

Additional file 1 **Complete list of genes that are specifically expressed in the airway epithelium**. List of genes with a significantly higher level of expression in the tracheal tissue compared with larval tissues minus trachea. Click here for file

Additional file 2 **Complete list of genes that are expressed in the airway epithelium**. All genes that consistently show a hybridization signal above backgound. Click here for file
