# Supplementary material for: Molecular architecture of the fruit fly's airway epithelial immune system
Source: BMC Genomics. 2008 Sep 29;9:446. doi: 10.1186/1471-2164-9-446 (PMC2566315; doi:10.1186/1471-2164-9-446)
Supplement: Additional file 1 — Complete list of genes that are specifically expressed in the airway epithelium. List of genes with a significantly higher level of expression in the tracheal tissue compared with larval tissues minus trachea. [file 1471-2164-9-446-S1.doc]

| Gene ID | STDEV | UniGene Name |
| --- | --- | --- |
| CG17347 | 0.64 | CG17347 |
| CG6186 | 0.22 | Transferrin 1 |
| CG8012 | 0.32 | CG8012 |
| CG18105 | 0.22 | Ecdysis triggering hormone |
| CG13640 | 0.38 | CG13640 |
| CG30154 | 0.50 | CG30154 |
| CG2520 | 0.25 | like-AP180 |
| CG30285 | 0.14 | CG30285 |
| CG7532 | 0.19 | CG7532 |
| CG32599 | 0.39 | CG32599 |
| CG10279 | 0.81 | Rm62 |
| CG30080 | 0.05 | CG30080 |
| CG6074 | 0.36 | CG6074 |
| CG11370 | 0.51 | CG11370 |
| CG4962 | 0.32 | CG4962 |
| CG9358 | 0.25 | NA |
| CG31997 | 0.12 | CG31997 |
| CG8511 | 0.05 | CG8511 |
| CG40486 | 0.42 |  |
| CG14572 | 0.30 | CG14572 |
| CG13067 | 0.53 | CG13067 |
| CG10112 | 0.32 | CG10112 |
| CG15251 | 0.23 | CG15251 |
| CG13117 | 0.23 | CG13117 |
| CG15201 | 0.32 | CG15201 |
| CG5867 | 0.21 | CG5867 |
| CG17190 | 0.05 | CG17190 |
| CG2139 | 0.08 | aralar1 |
| CG14984 | 0.05 | CG14984 |
| CG15080 | 0.11 | CG15080 |
| CG9020 | 0.42 | Arginyl-tRNA synthetase |
| CG33320 | 0.09 | NA |
| CG17218 | 0.17 | CG17218 |
| CG11390 | 0.08 | Ejaculatory bulb protein III |
| CG14903 | 0.49 | CG14903 |
| CG1780 | 0.21 | Imaginal disc growth factor 4 |
| CG7539 | 0.51 | Ecdysone-dependent gene 91 |
| CG13047 | 0.15 | CG13047 |
| CG7713 | 0.85 | CG7713 |
| CG2519 | 0.59 | CG2519 |
| CG14815 | 0.25 | CG14815 |
| CG3153 | 0.18 | CG3153 |
| CG3284 | 0.43 | RNA polymerase II 15kD subunit |
| CG18076 | 0.57 | short stop |
| CG30383 | 0.03 | CG30383 |
| CG14760 | 0.05 | CG14760 |
| CG1249 | 0.56 | CG1249 |
| CG30178 | 0.39 | CG30178 |
| CG4866 | 0.45 | CG4866 |
| CG14743 | 0.36 | CG14743 |
| CG14887 | 0.74 | Dihydrofolate reductase |
| CG9334 | 0.28 | Serine protease inhibitor 3 |
| CG1743 | 0.49 | Glutamine synthetase 2 |
| CG7637 | 0.68 | CG7637 |
| CG8409 | 0.54 | Suppressor of variegation 205 |
| CG10635 | 0.40 | CG10635 |
| CG7048 | 0.52 | CG7048 |
| CG8369 | 0.22 | CG8369 |
| CG8861 | 0.21 | CG8861 |
| CG8892 | 0.20 | CG8892 |
| CG13098 | 0.47 | mitochondrial ribosomal protein L51 |
| CG8526 | 0.17 | CG8526 |
| CG6302 | 0.11 | lethal (3) 01239 |
| CG8800 | 0.24 | CG8800 |
| CG14516 | 0.25 | NA |
| CG13068 | 0.19 | CG13068 |
| CG13224 | 0.13 | CG13224 |
| CG2875 | 0.20 | CG2875 |
| CG13628 | 0.17 | Rpb10 |
| CG10944 | 0.53 | Ribosomal protein S6 |
| CG15019 | 0.05 | CG15019 |
| CG12169 | 0.81 | CG12169 |
| CG13678 | 0.70 | CG13678 |
| CG9286 | 0.07 | CG9286 |
| CG30183 | 0.07 | CG30183 |
| CG3195 | 0.29 |  |
| CG3566 | 0.64 | CG3566 |
| CG15535 | 0.61 | CG15535 |
| CG3054 | 0.46 | lethal (2) k05819 |
| CG4337 | 0.20 | mitochondrial single stranded DNA-binding protein |
| CG5258 | 0.11 | NHP2 |
| CG30498 | 0.12 | boca |
| CG4665 | 0.52 | Dihydropteridine reductase |
| CG9336 | 0.12 | CG9336 |
| CG11797 | 0.30 | Odorant-binding protein 56a |
| CG14446 | 0.31 | CG14446 |
| CG16792 | 0.21 | Developmental embryonic B |
| CG3949 | 0.33 | hoi-polloi |
| CG4457 | 0.34 | Signal recognition particle protein 19 |
| CG5323 | 0.59 | CG5323 |
| CG13059 | 0.14 | CG13059 |
| CG14464 | 0.81 |  |
| CG6310 | 0.30 | CG6310 |
| CG15387 | 0.23 | CG15387 |
| CG33124 | 0.97 | CG33124 |
| CG6878 | 0.35 | CG6878 |
| CG8920 | 0.21 | CG8920 |
| CG7770 | 0.38 | CG7770 |
| CG8066 | 0.21 | CG8066 |
| CG5170 | 0.11 | Dodeca-satellite-binding protein 1 |
| CG9667 | 0.45 | CG9667 |
| CG14812 | 0.78 | NA |
| CG9277 | 0.21 | NA |
| CG4464 | 0.12 | Ribosomal protein S19a |
| CG11921 | 0.39 | forkhead domain 96Ca |
| CG9670 | 0.25 | falten |
| CG10679 | 0.34 | Nedd8 |
| CG11837 | 0.21 | CG11837 |
| CG7267 | 0.29 | CG7267 |
| CG8604 | 0.10 | Amphiphysin |
| CG10624 | 0.27 | sinuous |
| CG11279 | 0.40 | CG11279 |
| CG9077 | 0.33 | CG9077 |
| CG11271 | 0.72 | Ribosomal protein S12 |
| CG11885 | 0.12 | CG11885 |
| CG10603 | 0.59 | mitochondrial ribosomal protein L13 |
| CG12665 | 0.71 | Odorant-binding protein 8a |
| CG14096 | 0.52 | CG14096 |
| CG15083 | 0.04 | CG15083 |
| CG4584 | 0.46 | Deoxyuridine triphosphatase |
| CG11852 | 0.20 | CG11852 |
| CG10596 | 0.19 | Msr-110 |
| CG11985 | 0.29 | CG11985 |
| CG17776 | 0.07 | CG17776 |
| CG11883 | 0.75 | CG11883 |
| CG15353 | Jan 31 | CG15353 |
| CG5739 | 0.16 | CG5739 |
| CG9295 | 0.09 | CG9295 |
| CG11979 | 0.60 | Rpb5 |
| CG15012 | 0.25 | CG15012 |
| CG11482 | 0.16 | Mlh1 |
| CG32856 | 0.60 | CG32856 |
| CG14888 | 0.63 | CG14888 |
| CG3379 | 0.18 | Histone H4 replacement |
| CG16869 | 0.35 | Ance-2 |
| CG2811 | 0.43 | CG2811 |
| CG13049 | 0.45 | CG13049 |
| CG1516 | 0.32 | CG1516 |
| CG3161 | 0.51 |  |
| CG8918 | 0.48 | CG8918 |
| CG2330 | 0.46 | CG2330 |
| CG4494 | 0.25 | smt3 |
| CG3035 | 0.10 | carmine |
| CG30358 | 0.05 | CG30358 |
| CG7977 | 0.68 | Ribosomal protein L23A |
| CG9586 | 0.77 | CG9586 |
| CG12873 | 0.51 | CG12873 |
| CG3751 | 0.22 | Ribosomal protein S24 |
| CG7380 | 0.69 | NA |
| CG10418 | 0.13 | CG10418 |
| CG11858 | 0.25 | CG11858 |
| CG11859 | 0.03 | CG11859 |
| CG13403 | 0.53 | CG13403 |
| CG3595 | 0.15 | spaghetti squash |
| CG14894 | 0.69 | CG14894 |
| CG3450 | 0.45 | lethal (2) k03203 |
| CG6426 | 0.16 | CG6426 |
| CG16817 | 0.33 | CG16817 |
| CG17549 | 0.19 | CG17549 |
| CG1890 | 0.37 | CG1890 |
| CG32854 | 0.58 | mitochondrial ribosomal protein S21 |
| CG1019 | 0.10 | Muscle LIM protein at 84B |
| CG10219 | 0.09 | CG10219 |
| CG11901 | 0.32 | NA |
| CG13072 | 0.22 | PDCD-5 |
| CG9742 | 0.56 | CG9742 |
| CG10700 | 0.85 | CG10700 |
| CG8834 | 0.07 | CG8834 |
| CG9099 | 0.22 | CG9099 |
| CG8386 | 0.29 | CG8386 |
| CG30459 | 0.74 | CG30459 |
| CG4204 | 0.54 | Elongin B |
| CG9338 | 0.22 | CG9338 |
| CG5996 | 0.39 | NA |
| CG14359 | 0.90 | CG14359 |
| CG15191 | 0.11 | enhancer of yellow 2 |
| CG2901 | 0.05 | CG2901 |
| CR32314 | 0.88 |  |
| CG6770 | 0.54 | CG6770 |
| CG6459 | 0.15 | CG6459 |
| CG7224 | 0.48 | NA |
| CG7269 | 0.51 | Helicase at 25E |
| CG14447 | 0.95 | Glutamate receptor binding protein |
| CG18619 | 0.24 | CG18619 |
| CG30372 | 0.09 | CG30372 |
| CG6610 | 0.57 | CG6610 |
| CG11563 | 0.13 | CG11563 |
| CG13751 | 0.28 | CG13751 |
| CG9193 | 0.44 | mutagen-sensitive 209 |
| CG18001 | 0.05 |  |
| CG3224 | 0.63 | CG3224 |
| CG5161 | 0.42 | CG5161 |
| CG15881 | 0.27 | CG15881 |
| CG17896 | 0.50 | CG17896 |
| CG3242 | 0.35 | sister of odd and bowl |
| CG13044 | 0.43 | CG13044 |
| CG14977 | 0.15 | CG14977 |
| CG8781 | 0.53 | tsunagi |
| CG5676 | 0.41 | CG5676 |
| CG30105 | 0.17 | CG30105 |
| CG31184 | 0.30 | CG31184 |
| CG8498 | 0.17 | CG8498 |
| CG9075 | 0.51 | Eukaryotic initiation factor 4a |
| CG9730 | 0.29 | mitochondrial ribosomal protein L21 |
| CG1821 | 0.63 |  |
| CG32412 | 0.24 | CG32412 |
| CG10460 | 0.03 | crammer |
| CG4535 | 0.79 | FK506-binding protein FKBP59 |
| CG17765 | 0.57 | CG17765 |
| CG6056 | 0.42 | NA |
| CG6012 | 0.05 | CG6012 |
| CG1161 | 0.24 | CG1161 |
| CG15361 | 0.66 | Neuropeptide-like precursor 4 |
| CG30484 | 0.82 | CG30484 |
| CG5461 | 0.82 | bunched |
| CG40439 | 0.53 |  |
| CG11267 | 0.55 | CG11267 |
| CG12770 | 0.26 | Vps28 |
| CG15442 | 0.34 |  |
| CG8302 | 0.43 | Cyp4aa1 |
| CG8891 | 0.05 | CG8891 |
| CG30423 | 0.16 | CG30423 |
| CG3719 | 0.41 | CG3719 |
| CG12752 | 0.41 | NTF2-related export protein 1 |
| CG3226 | 0.21 | CG3226 |
| CG15003 | 0.68 | CG15003 |
| CG11218 | 0.26 | Odorant-binding protein 56d |
| CG11241 | 0.27 | CG11241 |
| CG1913 | 0.39 | NA |
| CG40045 | 0.51 |  |
| CG7917 | 0.19 | Nucleoplasmin |
| CG9812 | 0.20 | CG9812 |
| CG17059 | 0.28 | CG17059 |
| CG5864 | 0.41 | NA |
| CG7375 | 0.72 | CG7375 |
| CR40454 | 0.30 |  |
| CG10106 | 0.15 | Tetraspanin 42Ee |
| CG4264 | 0.36 | Heat shock protein cognate 4 |
| CG5119 | 0.27 | polyA-binding protein |
| CG5499 | 0.25 | Histone H2A variant |
| CG7073 | 0.40 | sar1 |
| CG10537 | 0.49 | Resistant to dieldrin |
| CG18767 | 0.54 | mitochondrial ribosomal protein L36 |
| CG32714 | 0.77 | CG32714 |
| CG8396 | 0.25 | Single stranded-binding protein c31A |
| CG13779 | 0.29 | CG13779 |
| CG18319 | 0.05 | bendless |
| CG5317 | 0.11 | CG5317 |
| CG8945 | 0.29 | CG8945 |
| CG9669 | 0.32 | CG9669 |
| CG10751 | 0.57 | roadblock |
| CG13298 | 0.18 | CG13298 |
| CG5057 | 0.51 | Mediator complex subunit 10 |
| CG14865 | 0.29 | lethal (3) neo43 |
| CG10527 | 0.37 | CG10527 |
| CG13364 | 0.05 | CG13364 |
| CG14757 | 0.31 | CG14757 |
| CG14981 | 0.35 | maggie |
| CG3129 | 0.10 | Rab-related protein 4 |
| CG5730 | 0.52 | Annexin IX |
| CG1475 | 0.15 | Ribosomal protein L13A |
| CG1660 | 0.56 | Tim9a |
| CG8857 | 0.67 | Ribosomal protein S11 |
| CG14715 | 0.05 | CG14715 |
| CG2813 | 0.23 | CG2813 |
| CG4605 | 0.48 | Accessory gland-specific peptide 32CD |
| CG4928 | 0.58 | NA |
| CG7038 | 0.07 | mitochondrial ribosomal protein L30 |
| CG8048 | 0.92 | Vacuolar H |
| CG9353 | 0.12 | mitochondrial ribosomal protein L54 |
| CG10117 | 0.17 | tout-velu |
| CG11781 | 0.89 | CG11781 |
| CG13993 | 0.05 | CG13993 |
| CG13994 | 0.23 | CG13994 |
| CG15210 | 0.15 | CG15210 |
| CG15730 | 0.36 | CG15730 |
| CG30436 | 0.80 | CG30436 |
| CG1081 | 0.32 | NA |
| CG1151 | 0.42 | Osiris 6 |
| CG3395 | 0.36 | Ribosomal protein S9 |
| CG10306 | 0.20 | CG10306 |
| CG11077 | 0.14 | CG11077 |
| CG15697 | 0.17 | Ribosomal protein S30 |
| CG32413 | 0.43 | CG32413 |
| CG6998 | 0.09 | cut up |
| CG4004 | 0.60 | CG4004 |
| CG7911 | 0.21 | CG7911 |
| CR30425 | 0.36 |  |
| CG9856 | 0.18 | Protein tyrosine phosphatase-ERK/Enhancer of Ras1 |
| CG8542 | 0.54 | Heat shock protein cognate 5 |
| CG12443 | 0.32 | thisbe |
| CG14104 | 0.14 | CG14104 |
| CG14966 | 0.35 | CG14966 |
| CG3204 | 0.46 | Ras-associated protein 2-like |
| CG4820 | 0.65 | CG4820 |
| CG5277 | 0.25 | Intronic Protein 259 |
| CG8156 | 0.42 | ADP ribosylation factor 51F |
| CG18096 | 0.40 | Thiolester containing protein I |
| CG5788 | 0.28 | Ubiquitin conjugating enzyme 10 |
| CG1242 | 0.33 | Heat shock protein 83 |
| CG10373 | 0.27 | CG10373 |
| CG11906 | 0.35 | CG11906 |
| CG18081 | 0.54 | CG18081 |
| CG8827 | 0.25 | Angiotensin converting enzyme |
| CG12775 | 0.57 | Ribosomal protein L21 |
| CG13545 | 0.49 | CG13545 |
| CG15693 | 0.54 | Ribosomal protein S20 |
| CG12358 | 0.30 | polyA-binding protein interacting protein 2 |
| CG12848 | 0.50 | CG12848 |
| CG13674 | 0.27 | CG13674 |
| CG3661 | 0.05 | Ribosomal protein L23 |
| CG4647 | 0.45 | mitochondrial ribosomal protein L49 |
| CG4760 | 0.77 | boule |
| CG7933 | 0.35 | janus A |
| CG8860 | 0.35 | CG8860 |
| CG15506 | 0.25 | CG15506 |
| CG1728 | 0.13 | Tim8 |
| CG2168 | 0.10 | Ribosomal protein S3A |
| CG3420 | 0.43 | CG3420 |
| CG8900 | 0.10 | Ribosomal protein S18 |
| CG10144 | 0.13 | CG10144 |
| CG10938 | 0.10 | Proteasome |
| CG1163 | 0.07 | RNA polymerase II 18kD subunit |
| CG33198 | 0.61 | presenilin enhancer |
| CG40218 | 0.44 |  |
| CG8472 | 0.12 | Calmodulin |
| CG3024 | 0.15 | torp4a |
| CG31450 | 0.33 | mitochondrial ribosomal protein S18A |
| CG33184 | 0.35 |  |
| CG5855 | 0.25 | cornichon |
| CG7622 | 0.65 | Ribosomal protein L36 |
| CG12304 | 0.18 | CG12304 |
| CG13393 | 0.46 | CG13393 |
| CG15717 | 0.18 | CG15717 |
| CG16982 | 0.07 | Roc1a |
| CG31705 | 0.35 | CG31705 |
| CG32442 | 0.56 | CG32442 |
| CG6712 | 0.23 | CG6712 |
| CG11790 | 0.18 | CG11790 |
| CG2219 | 0.41 | CG2219 |
| CG6253 | 0.20 | Ribosomal protein L14 |
| CG6779 | 0.11 | Ribosomal protein S3 |
| CG8338 | 0.10 | mitochondrial ribosomal protein S16 |
| CG12214 | 0.31 | CG12214 |
| CG17680 | 0.13 | CG17680 |
| CG2845 | 0.72 | pole hole |
| CG31196 | 0.19 | NA |
| CG8331 | 0.31 | CG8331 |
| CR40456 | 0.48 |  |
| CG13042 | 0.21 | CG13042 |
| CG3034 | 0.41 | Mediator complex subunit 22 |
| CG7823 | 0.62 | RhoGDI |
| CG10652 | 0.34 |  |
| CG14566 | 0.12 | CG14566 |
| CG30173 | 0.14 | Syntaxin Interacting Protein 1 |
| CG4863 | 0.13 | Ribosomal protein L3 |
| CG9548 | 0.10 | CG9548 |
| CG12324 | 0.22 | Ribosomal protein S15Ab |
| CG4651 | 0.15 | Ribosomal protein L13 |
| CG5826 | 0.20 | Peroxiredoxin 5037 |
| CG8268 | 0.30 | CG8268 |
| CG10673 | 0.21 | CG10673 |
| CG12491 | 0.43 | CG12491 |
| CG7354 | 0.21 | mitochondrial ribosomal protein S26 |
| CG11024 | 0.30 |  |
| CG6465 | 0.29 | CG6465 |
| CG9847 | 0.08 | Fkbp13 |
| CG9878 | 0.21 | Tim10 |
| CG4944 | 0.21 | ciboulot |
| CG5210 | 0.46 | Chitinase-like |
| CG6513 | 0.17 | endosulfine |
| CG6988 | 0.23 | Protein disulfide isomerase |
| CG8372 | 0.12 | CG8372 |
| CG9796 | 0.24 | CG9796 |
| CG11922 | 0.07 | forkhead domain 96Cb |
| CG12352 | 0.24 | separation anxiety |
| CG13880 | 0.12 | mitochondrial ribosomal protein L17 |
| CG17737 | 0.07 | CG17737 |
| CG1943 | 0.08 | CG1943 |
| CG32440 | 0.15 | CG32440 |
| CG5446 | 0.11 | CG5446 |
| CG5827 | 0.17 | Ribosomal protein L37A |
| CG7484 | 0.08 | CG7484 |
| CG16765 | 0.54 | lethal (3) 10615 |
| CG30460 | 0.50 | CG30460 |
| CG5029 | 0.21 | S-adenosylmethionine decarboxylase |
| CG6025 | 0.40 | Arflike at 72A |
| CG8882 | 0.07 | Trip1 |
| CG9035 | 0.27 | NA |
| CG9344 | 0.07 | CG9344 |
| CG17521 | 0.41 | Qm |
| CG7196 | 0.50 | CG7196 |
| CG2852 | 0.08 | CG2852 |
| CG32736 | 0.48 | CG32736 |
| CG3887 | 0.08 | CG3887 |
| CG14184 | 0.37 | CG14184 |
| CG15715 | 0.25 | CG15715 |
| CG2163 | 0.28 | Pabp2 |
| CG10473 | 0.46 | CG10473 |
| CG13200 | 0.45 | CG13200 |
| CG15168 | 0.15 | CG15168 |
| CG2767 | 0.05 | CG2767 |
| CG32693 | 0.53 | Gr9a |
| CG4800 | 0.33 | Translationally controlled tumor protein |
| CG9410 | 0.51 | CG9410 |
| CG14985 | 0.32 | CG14985 |
| CG16936 | 0.48 | NA |
| CG18177 | 0.42 | CG18177 |
| CG32267 | 0.31 | CG32267 |
| CG4897 | 0.07 | Ribosomal protein L7 |
| CG8332 | 0.07 | Ribosomal protein S15 |
| CG9740 | 0.13 | CG9740 |
