# Supplementary material for: Molecular architecture of the fruit fly's airway epithelial immune system
Source: BMC Genomics. 2008 Sep 29;9:446. doi: 10.1186/1471-2164-9-446 (PMC2566315; doi:10.1186/1471-2164-9-446)
Supplement: Additional file 2 — Complete list of genes that are expressed in the airway epithelium. All genes that consistently show a hybridization signal above backgound. [file 1471-2164-9-446-S2.doc]

CG10005

CG10009

CG10021

CG10036

CG10037

CG10039

CG10045

CG10055

CG10060

CG10067

CG1007

CG10071

CG10075

CG10077

CG10091

CG10106

CG10107

CG10112

CG10116

CG10117

CG10118

CG10119

CG10120

CG10126

CG10128

CG10130

CG10139

CG10140

CG10144

CG10145

CG10146

CG10149

CG10153

CG10154

CG10159

CG10161

CG10166

CG10170

CG1019

CG10200

CG10206

CG10208

CG10210

CG10214

CG10217

CG10219

CG10230

CG10238

CG1024

CG10242

CG10243

CG10263

CG10279

CG1028

CG10281

CG10287

CG10293

CG10297

CG10300

CG10301

CG10302

CG10303

CG10305

CG10306

CG10311

CG10315

CG10327

CG10333

CG10334

CG10335

CG10339

CG10340

CG10343

CG10347

CG10348

CG10364

CG10370

CG10371

CG10373

CG10375

CG10392

CG10395

CG10405

CG10406

CG10418

CG10423

CG10424

CG10433

CG10445

CG10447

CG10460

CG10465

CG10466

CG10467

CG10470

CG10472

CG10473

CG10475

CG10477

CG10481

CG10483

CG10484

CG10497

CG10498

CG10501

CG10504

CG10514

CG10520

CG10524

CG10527

CG10528

CG10529

CG10530

CG10531

CG10533

CG10536

CG10537

CG10538

CG10540

CG10543

CG10545

CG10550

CG10562

CG10570

CG10574

CG10576

CG1058

CG10582

CG10585

CG10588

CG10589

CG1059

CG10596

CG10598

CG10601

CG10603

CG10617

CG10622

CG10624

CG10625

CG10627

CG10630

CG10631

CG10633

CG10635

CG10638

CG10640

CG1065

CG10650

CG10652

CG10664

CG10672

CG10673

CG10674

CG10679

CG10682

CG10683

CG10685

CG10688

CG10691

CG10692

CG10693

CG10700

CG10719

CG10725

CG10728

CG10732

CG10733

CG10746

CG10751

CG10753

CG10756

CG10757

CG1077

CG10781

CG10788

CG10804

CG10806

CG1081

CG10810

CG10811

CG10825

CG10827

CG10837

CG1084

CG10845

CG10846

CG10849

CG10851

CG10853

CG10858

CG10861

CG10863

CG10866

CG1088

CG10888

CG10898

CG10907

CG10908

CG1091

CG10910

CG10911

CG10912

CG10918

CG10922

CG10938

CG10939

CG10944

CG10953

CG10954

CG10962

CG10964

CG10978

CG10979

CG10992

CG11001

CG11015

CG11024

CG11027

CG11035

CG11042

CG11051

CG11059

CG1107

CG11076

CG11077

CG1109

CG11106

CG11113

CG1112

CG11122

CG11134

CG11137

CG11139

CG11142

CG11143

CG11151

CG11154

CG11155

CG11158

CG11166

CG11175

CG11177

CG11181

CG11217

CG11218

CG11230

CG11235

CG11241

CG11242

CG11246

CG11255

CG11258

CG11266

CG11267

CG11268

CG11271

CG11276

CG11278

CG11279

CG11280

CG11281

CG11301

CG11306

CG11307

CG11309

CG11317

CG1134

CG11342

CG11347

CG11350

CG11352

CG11370

CG11372

CG11377

CG11390

CG1140

CG11400

CG11403

CG11407

CG11413

CG11415

CG1142

CG11421

CG11444

CG11447

CG11454

CG11455

CG11458

CG11466

CG1147

CG11470

CG11471

CG11482

CG11488

CG11491

CG11500

CG11501

CG1151

CG11522

CG11525

CG11529

CG11555

CG11562

CG11563

CG11567

CG11579

CG11581

CG11589

CG11590

CG11606

CG11607

CG1161

CG11611

CG11614

CG1162

CG11624

CG11629

CG1163

CG11635

CG11637

CG11638

CG11642

CG1165

CG11650

CG11656

CG11661

CG11671

CG11672

CG11678

CG11686

CG11699

CG11700

CG11709

CG11742

CG11752

CG11753

CG11761

CG11768

CG11777

CG11779

CG11781

CG11783

CG11784

CG11785

CG11786

CG11788

CG1179

CG11790

CG11791

CG11793

CG11797

CG11798

CG11804

CG11820

CG11824

CG11828

CG11833

CG11837

CG11839

CG11840

CG11844

CG11849

CG11851

CG11852

CG11853

CG11854

CG11858

CG11859

CG11874

CG11881

CG11883

CG11885

CG11888

CG11900

CG11901

CG11905

CG11906

CG11911

CG11912

CG11920

CG11921

CG11922

CG11958

CG11979

CG11981

CG11982

CG11985

CG11989

CG11990

CG11999

CG1200

CG12000

CG12004

CG12012

CG12013

CG12038

CG12051

CG12052

CG12055

CG12057

CG12079

CG12082

CG12085

CG12091

CG12101

CG12104

CG12107

CG12116

CG12117

CG12121

CG12123

CG12127

CG12129

CG12130

CG12131

CG12135

CG12139

CG1214

CG12141

CG12147

CG12155

CG12159

CG12169

CG12171

CG12173

CG12203

CG12209

CG12210

CG12212

CG12214

CG12217

CG12220

CG12233

CG12262

CG12265

CG12276

CG12279

CG12284

CG12297

CG12304

CG12310

CG12323

CG12324

CG12333

CG12343

CG12346

CG12348

CG12350

CG12352

CG12355

CG12357

CG12358

CG12363

CG12367

CG12369

CG12372

CG12373

CG12379

CG12384

CG12386

CG12388

CG12389

CG12393

CG1240

CG12400

CG12404

CG1242

CG12424

CG12425

CG12443

CG12449

CG12452

CG12454

CG1249

CG12491

CG12501

CG12519

CG12522

CG12541

CG12546

CG12559

CG12564

CG12567

CG12602

CG12617

CG12643

CG12656

CG12665

CG12676

CG12699

CG12724

CG1273

CG12730

CG12734

CG12737

CG1274

CG12744

CG12749

CG1275

CG12752

CG12756

CG1276

CG12763

CG12765

CG12766

CG12770

CG12775

CG12780

CG12782

CG12785

CG12789

CG12797

CG12811

CG12819

CG12825

CG12832

CG12840

CG12845

CG12846

CG12847

CG12848

CG12859

CG12868

CG12869

CG12873

CG12874

CG12876

CG12879

CG12895

CG12918

CG12929

CG12934

CG12935

CG12938

CG12948

CG12954

CG12975

CG1298

CG12985

CG1299

CG13014

CG13018

CG13037

CG13038

CG13042

CG13044

CG13046

CG13047

CG13049

CG13059

CG13063

CG13066

CG13067

CG13068

CG13069

CG1307

CG13072

CG13075

CG13095

CG13096

CG13098

CG13102

CG1311

CG13110

CG13116

CG13117

CG13124

CG13160

CG1317

CG1318

CG1319

CG13190

CG1320

CG13200

CG13210

CG13211

CG13220

CG13224

CG13230

CG13255

CG13258

CG13280

CG13281

CG13284

CG13298

CG13311

CG13315

CG13319

CG13321

CG13323

CG13324

CG13335

CG13339

CG13361

CG13364

CG13374

CG13377

CG13379

CG13389

CG13391

CG13393

CG13396

CG13403

CG13410

CG13415

CG13418

CG13434

CG1345

CG13460

CG13475

CG13484

CG1349

CG13504

CG13510

CG13533

CG13545

CG13551

CG13567

CG13585

CG1359

CG13601

CG13603

CG13608

CG13609

CG13623

CG13628

CG13630

CG13640

CG13641

CG13663

CG13674

CG13678

CG1368

CG13689

CG13693

CG13695

CG13698

CG13727

CG13746

CG13751

CG13773

CG13779

CG13788

CG1380

CG13807

CG1381

CG13822

CG13840

CG13849

CG1385

CG13860

CG13865

CG13867

CG13879

CG13880

CG13887

CG13890

CG13901

CG13905

CG13912

CG13914

CG13919

CG13922

CG13923

CG13926

CG13941

CG13947

CG13951

CG13962

CG13990

CG13993

CG13994

CG14007

CG14028

CG14029

CG1404

CG14045

CG14048

CG14057

CG14066

CG14084

CG14087

CG14096

CG14102

CG14104

CG14132

CG14145

CG14147

CG1416

CG14181

CG14184

CG14199

CG14205

CG14206

CG14207

CG14210

CG14214

CG14215

CG14222

CG14229

CG14235

CG14237

CG14240

CG14254

CG14258

CG14265

CG14266

CG14270

CG14271

CG14283

CG14286

CG1429

CG14290

CG14299

CG14302

CG14307

CG14322

CG14341

CG14359

CG14365

CG14394

CG14401

CG14407

CG14413

CG14424

CG1443

CG14430

CG14434

CG14437

CG14446

CG14447

CG14450

CG14463

CG14464

CG14476

CG14477

CG1448

CG14480

CG14482

CG14483

CG14487

CG1449

CG14500

CG14511

CG14512

CG14516

CG14520

CG14543

CG14548

CG14550

CG14566

CG14567

CG14572

CG14577

CG1458

CG14598

CG14619

CG14643

CG14647

CG14648

CG14661

CG14671

CG1468

CG14683

CG1469

CG14711

CG14715

CG14716

CG14724

CG14743

CG14745

CG1475

CG14750

CG14752

CG14757

CG14760

CG14767

CG14777

CG14782

CG14792

CG14795

CG14802

CG14806

CG14812

CG14815

CG14816

CG14817

CG14818

CG14821

CG14830

CG14840

CG14852

CG1486

CG14865

CG14872

CG14884

CG14887

CG14888

CG14894

CG14898

CG14902

CG14903

CG14906

CG14907

CG14909

CG14915

CG14933

CG14938

CG14939

CG14957

CG14966

CG14977

CG14981

CG14984

CG14985

CG14996

CG14997

CG14998

CG14999

CG15000

CG15003

CG15011

CG15012

CG15014

CG15016

CG15019

CG15027

CG15030

CG15043

CG15046

CG15065

CG1507

CG15073

CG15080

CG15081

CG15083

CG15093

CG15096

CG15098

CG15101

CG15102

CG15119

CG15127

CG15133

CG15152

CG15155

CG1516

CG15168

CG1518

CG1519

CG15191

CG15201

CG15204

CG15210

CG15211

CG15213

CG15220

CG15224

CG15237

CG1524

CG15250

CG15251

CG15254

CG15261

CG15266

CG1527

CG15276

CG15277

CG1528

CG15281

CG15282

CG15293

CG15295

CG15304

CG15308

CG1531

CG15312

CG1532

CG15337

CG15347

CG15353

CG15357

CG15361

CG15362

CG15367

CG15369

CG15386

CG15387

CG15396

CG15398

CG15403

CG15408

CG15422

CG15423

CG15427

CG15432

CG15442

CG15457

CG15459

CG15461

CG15472

CG15477

CG1548

CG15481

CG15497

CG15504

CG15506

CG15525

CG15532

CG15535

CG15536

CG1554

CG15593

CG15629

CG15633

CG15645

CG15653

CG15654

CG15673

CG15675

CG15684

CG1569

CG15690

CG15693

CG15697

CG15701

CG15707

CG1571

CG15715

CG15717

CG1572

CG15728

CG15730

CG15735

CG15736

CG15741

CG15742

CG15743

CG15753

CG15756

CG15757

CG15760

CG15764

CG15766

CG1577

CG1578

CG15780

CG15784

CG15800

CG15812

CG15818

CG15825

CG15829

CG1583

CG15835

CG15845

CG15848

CG15863

CG1587

CG15871

CG15878

CG15881

CG15882

CG15884

CG1591

CG15916

CG15917

CG15922

CG15930

CG1598

CG1599

CG1600

CG1620

CG1621

CG1633

CG1634

CG1636

CG1639

CG1640

CG1643

CG1648

CG1651

CG1657

CG1659

CG1660

CG16704

CG16707

CG16711

CG16712

CG16713

CG16724

CG16725

CG16727

CG1674

CG16741

CG16743

CG16753

CG1676

CG16765

CG16775

CG16784

CG16792

CG16793

CG16799

CG16812

CG16817

CG16858

CG16869

CG16892

CG16901

CG16914

CG16916

CG16926

CG16935

CG16936

CG16944

CG16953

CG16957

CG16969

CG1698

CG16982

CG16983

CG16985

CG16986

CG16996

CG16997

CG1702

CG17023

CG17026

CG17029

CG1703

CG17032

CG17052

CG17058

CG17059

CG1707

CG17082

CG17107

CG17108

CG17117

CG17124

CG17134

CG17136

CG17143

CG17145

CG1715

CG17173

CG17190

CG17202

CG17218

CG17249

CG17266

CG17271

CG17272

CG17273

CG17278

CG1728

CG17280

CG17282

CG17293

CG17294

CG17295

CG17325

CG17327

CG17330

CG17331

CG17332

CG17333

CG17334

CG17343

CG17347

CG17358

CG1738

CG17385

CG17397

CG1742

CG17420

CG1743

CG17440

CG1746

CG17475

CG17486

CG17489

CG1749

CG17493

CG17494

CG17498

CG1750

CG17508

CG1751

CG17510

CG17520

CG17521

CG17522

CG17524

CG17527

CG1753

CG17531

CG17533

CG17534

CG17549

CG17556

CG17567

CG17571

CG17574

CG17597

CG17600

CG17618

CG17633

CG17639

CG17654

CG17680

CG17686

CG17691

CG1771

CG17715

CG1772

CG17721

CG17734

CG17737

CG17753

CG17765

CG17776

CG1780

CG17814

CG17828

CG17834

CG17841

CG1786

CG17870

CG1789

CG17894

CG17896

CG17903

CG17906

CG17914

CG17919

CG17921

CG17922

CG17927

CG17931

CG17943

CG17949

CG17950

CG17956

CG17988

CG1799

CG17991

CG17996

CG18000

CG18001

CG18009

CG18023

CG18030

CG18066

CG18076

CG18081

CG18096

CG18105

CG1812

CG18124

CG1814

CG18140

CG18143

CG18145

CG18174

CG18176

CG18177

CG18179

CG18180

CG18188

CG1821

CG18211

CG18212

CG1822

CG18228

CG1824

CG18255

CG18268

CG18290

CG18294

CG18315

CG18316

CG18319

CG18321

CG18332

CG18335

CG18343

CG18348

CG18349

CG18358

CG1836

CG1837

CG18410

CG18416

CG18428

CG18437

CG1844

CG18444

CG1845

CG18466

CG18495

CG18505

CG18508

CG18518

CG18525

CG18547

CG18548

CG18553

CG18554

CG18561

CG1859

CG18591

CG18593

CG18594

CG18600

CG18619

CG18624

CG1864

CG18648

CG18649

CG1865

CG18681

CG1871

CG18731

CG18740

CG18767

CG18773

CG18777

CG18779

CG18783

CG18787

CG18803

CG18809

CG18811

CG18815

CG18816

CG18817

CG1883

CG1884

CG18853

CG18854

CG1890

CG1891

CG1893

CG1896

CG1901

CG1902

CG1906

CG1913

CG1921

CG1935

CG1939

CG1943

CG1957

CG1961

CG1963

CG1967

CG1968

CG1980

CG1983

CG1989

CG1998

CG2013

CG2014

CG2021

CG2033

CG2043

CG2046

CG2050

CG2054

CG2063

CG2064

CG2071

CG2079

CG2099

CG2101

CG2104

CG2109

CG2116

CG2139

CG2140

CG2145

CG2147

CG2152

CG2163

CG2168

CG2185

CG2200

CG2202

CG2207

CG2210

CG2216

CG2219

CG2227

CG2229

CG2238

CG2245

CG2248

CG2249

CG2262

CG2275

CG2297

CG2330

CG2331

CG2342

CG2358

CG2397

CG2444

CG2467

CG2493

CG2505

CG2508

CG2512

CG2519

CG2520

CG2522

CG2525

CG2530

CG2617

CG2621

CG2656

CG2668

CG2669

CG2674

CG2677

CG2699

CG2720

CG2727

CG2746

CG2767

CG2789

CG2803

CG2807

CG2811

CG2813

CG2816

CG2827

CG2845

CG2846

CG2852

CG2855

CG2859

CG2862

CG2875

CG2890

CG2901

CG2915

CG2922

CG2934

CG2937

CG2944

CG2947

CG2956

CG2960

CG2961

CG2968

CG2974

CG2976

CG2986

CG2998

CG30000

CG30005

CG30007

CG3002

CG30028

CG30029

CG3003

CG30034

CG30044

CG30046

CG30051

CG30052

CG30077

CG30080

CG30092

CG30094

CG30100

CG30105

CG30108

CG30109

CG3011

CG30121

CG30122

CG30148

CG30151

CG30154

CG30159

CG3016

CG30160

CG30161

CG30163

CG30169

CG30171

CG30172

CG30173

CG30178

CG30179

CG30183

CG30185

CG30188

CG3019

CG30195

CG30196

CG30197

CG3021

CG30217

CG30219

CG30222

CG3024

CG30259

CG30265

CG30268

CG30274

CG30275

CG30277

CG30278

CG30279

CG30281

CG30285

CG3029

CG30291

CG30293

CG30337

CG30338

CG3034

CG30349

CG3035

CG30357

CG30358

CG30371

CG30372

CG30373

CG30379

CG30380

CG30382

CG30383

CG3039

CG30392

CG30409

CG30410

CG30411

CG30415

CG30417

CG30423

CG30427

CG30428

CG30430

CG30436

CG30437

CG30457

CG30459

CG30460

CG30472

CG30476

CG30484

CG30491

CG30497

CG30498

CG30499

CG3050

CG30501

CG30503

CG3054

CG3056

CG3058

CG3066

CG3074

CG3083

CG31000

CG31005

CG31009

CG31012

CG31018

CG31041

CG31079

CG31086

CG31109

CG31121

CG31126

CG31142

CG31148

CG31170

CG31184

CG31196

CG31202

CG31226

CG31237

CG31267

CG3127

CG31289

CG3129

CG31292

CG31293

CG31300

CG31301

CG31304

CG31305

CG3131

CG31313

CG31314

CG31317

CG31320

CG31324

CG31337

CG31344

CG31349

CG31352

CG31360

CG31362

CG3140

CG31410

CG31421

CG31439

CG31450

CG31460

CG31469

CG31472

CG31478

CG31483

CG31488

CG31509

CG31522

CG31526

CG3153

CG31535

CG31548

CG31549

CG31605

CG31606

CG3161

CG31613

CG3162

CG31624

CG31641

CG31648

CG31649

CG31692

CG31705

CG31710

CG31715

CG31717

CG31723

CG31729

CG31740

CG31743

CG31749

CG31758

CG31760

CG31764

CG31775

CG31783

CG31789

CG31794

CG31800

CG31820

CG31826

CG3183

CG31839

CG31842

CG31855

CG3186

CG31864

CG31872

CG31873

CG31893

CG31901

CG3191

CG31917

CG31922

CG3195

CG31950

CG31957

CG31974

CG31990

CG31997

CG3200

CG3201

CG32022

CG32025

CG3203

CG32031

CG32036

CG32038

CG32039

CG3204

CG32041

CG32042

CG32068

CG32069

CG32071

CG32079

CG32092

CG32109

CG32137

CG3214

CG32147

CG32163

CG32174

CG32177

CG32189

CG32198

CG32202

CG32208

CG32212

CG32217

CG32230

CG3224

CG32253

CG3226

CG32264

CG32266

CG32267

CG32276

CG32278

CG32280

CG32284

CG32302

CG32307

CG32318

CG32368

CG32398

CG32399

CG32402

CG32405

CG32407

CG32409

CG32410

CG32412

CG32413

CG32417

CG32418

CG3242

CG32428

CG3244

CG32440

CG32441

CG32442

CG32446

CG32448

CG32458

CG32469

CG3249

CG32491

CG32512

CG32530

CG32531

CG32542

CG32550

CG32564

CG32581

CG32590

CG32599

CG3262

CG32626

CG3264

CG32640

CG32645

CG3265

CG32656

CG32667

CG3267

CG32672

CG32687

CG32693

CG32708

CG32714

CG32736

CG32737

CG32741

CG32781

CG32797

CG32816

CG32817

CG3283

CG32830

CG3284

CG32850

CG32854

CG32856

CG3292

CG32920

CG32954

CG32956

CG33002

CG33009

CG3303

CG3304

CG33045

CG3305

CG33051

CG33070

CG33071

CG33096

CG33104

CG33105

CG33111

CG33113

CG33116

CG33124

CG33127

CG33128

CG33129

CG3314

CG33144

CG33149

CG33151

CG33162

CG33169

CG33170

CG33175

CG33178

CG33184

CG33188

CG33189

CG33192

CG33196

CG33198

CG3320

CG3321

CG33214

CG33217

CG3322

CG33228

CG33229

CG33254

CG33255

CG33261

CG33275

CG33276

CG3329

CG33292

CG33293

CG33295

CG33296

CG33297

CG33306

CG33307

CG33317

CG33320

CG33321

CG33330

CG33336

CG33339

CG33349

CG3337

CG3338

CG33456

CG33463

CG33466

CG33469

CG33477

CG33478

CG33479

CG3348

CG33483

CG33492

CG33493

CG33494

CG33495

CG3350

CG33502

CG33503

CG3351

CG3352

CG3359

CG3365

CG3379

CG3395

CG3397

CG3402

CG3403

CG3410

CG3413

CG3415

CG3420

CG3421

CG3422

CG3431

CG3434

CG3440

CG3446

CG3448

CG3450

CG3455

CG3483

CG3501

CG3509

CG3527

CG3541

CG3558

CG3560

CG3564

CG3566

CG3584

CG3594

CG3595

CG3604

CG3609

CG3612

CG3616

CG3619

CG3621

CG3624

CG3625

CG3633

CG3652

CG3656

CG3661

CG3662

CG3663

CG3664

CG3683

CG3690

CG3699

CG3700

CG3710

CG3712

CG3717

CG3719

CG3725

CG3731

CG3746

CG3751

CG3752

CG3756

CG3760

CG3762

CG3763

CG3767

CG3772

CG3776

CG3777

CG3781

CG3782

CG3800

CG3814

CG3817

CG3831

CG3832

CG3847

CG3850

CG3853

CG3868

CG3875

CG3881

CG3887

CG3891

CG3902

CG3907

CG3922

CG3923

CG3924

CG3939

CG3943

CG3944

CG3948

CG3949

CG3964

CG3967

CG3987

CG3988

CG3989

CG3991

CG3997

CG40002

CG4004

CG40042

CG40045

CG40049

CG4005

CG40050

CG4006

CG4008

CG40084

CG40100

CG40123

CG40127

CG4013

CG40166

CG40181

CG4019

CG40196

CG40218

CG40228

CG4025

CG4026

CG4027

CG40293

CG40305

CG4035

CG4036

CG40370

CG40381

CG4043

CG40439

CG4045

CG40451

CG4046

CG40467

CG40486

CG40498

CG4053

CG4065

CG4070

CG4071

CG4074

CG4079

CG4086

CG4094

CG4097

CG4098

CG4101

CG4108

CG4109

CG4111

CG41128

CG41133

CG4122

CG4123

CG4128

CG4140

CG4143

CG4147

CG4153

CG4162

CG4164

CG4169

CG4178

CG4181

CG4183

CG4186

CG4195

CG4204

CG4205

CG4207

CG4210

CG4212

CG4233

CG4236

CG4247

CG4254

CG4264

CG4265

CG4266

CG4276

CG4278

CG4279

CG4299

CG4306

CG4307

CG4312

CG4325

CG4337

CG4338

CG4360

CG4362

CG4363

CG4364

CG4367

CG4371

CG4373

CG4376

CG4379

CG4381

CG4389

CG4390

CG4406

CG4412

CG4420

CG4422

CG4427

CG4437

CG4443

CG4446

CG4447

CG4455

CG4457

CG4461

CG4463

CG4464

CG4475

CG4482

CG4486

CG4491

CG4494

CG4510

CG4523

CG4528

CG4533

CG4535

CG4554

CG4559

CG4584

CG4592

CG4594

CG4598

CG4600

CG4602

CG4605

CG4609

CG4618

CG4621

CG4627

CG4634

CG4646

CG4647

CG4651

CG4653

CG4662

CG4665

CG4678

CG4686

CG4692

CG4696

CG4698

CG4712

CG4716

CG4726

CG4729

CG4730

CG4734

CG4746

CG4760

CG4769

CG4780

CG4789

CG4798

CG4800

CG4802

CG4820

CG4830

CG4843

CG4849

CG4858

CG4863

CG4866

CG4869

CG4884

CG4886

CG4893

CG4897

CG4898

CG4904

CG4912

CG4914

CG4916

CG4918

CG4924

CG4928

CG4930

CG4933

CG4935

CG4944

CG4946

CG4951

CG4957

CG4962

CG4968

CG4974

CG4980

CG4994

CG5001

CG5012

CG5014

CG5017

CG5021

CG5023

CG5025

CG5029

CG5045

CG5056

CG5057

CG5058

CG5065

CG5067

CG5072

CG5084

CG5104

CG5108

CG5110

CG5116

CG5119

CG5125

CG5134

CG5148

CG5161

CG5162

CG5163

CG5164

CG5166

CG5170

CG5172

CG5174

CG5177

CG5183

CG5184

CG5189

CG5191

CG5193

CG5194

CG5201

CG5203

CG5206

CG5210

CG5214

CG5219

CG5220

CG5224

CG5242

CG5246

CG5258

CG5266

CG5268

CG5271

CG5277

CG5282

CG5289

CG5304

CG5310

CG5313

CG5317

CG5323

CG5327

CG5330

CG5333

CG5335

CG5343

CG5345

CG5346

CG5355

CG5357

CG5362

CG5378

CG5380

CG5382

CG5385

CG5390

CG5391

CG5395

CG5399

CG5413

CG5417

CG5434

CG5442

CG5446

CG5450

CG5452

CG5454

CG5461

CG5474

CG5476

CG5479

CG5482

CG5486

CG5492

CG5495

CG5497

CG5499

CG5502

CG5506

CG5515

CG5516

CG5517

CG5518

CG5525

CG5532

CG5537

CG5547

CG5548

CG5550

CG5558

CG5562

CG5575

CG5589

CG5590

CG5596

CG5604

CG5605

CG5630

CG5651

CG5652

CG5676

CG5677

CG5680

CG5703

CG5708

CG5727

CG5730

CG5733

CG5734

CG5738

CG5739

CG5741

CG5757

CG5765

CG5767

CG5770

CG5771

CG5773

CG5783

CG5784

CG5788

CG5792

CG5802

CG5804

CG5809

CG5812

CG5820

CG5825

CG5826

CG5827

CG5835

CG5842

CG5844

CG5848

CG5849

CG5851

CG5854

CG5855

CG5860

CG5861

CG5862

CG5864

CG5867

CG5869

CG5872

CG5880

CG5883

CG5885

CG5887

CG5889

CG5896

CG5903

CG5911

CG5915

CG5920

CG5932

CG5934

CG5935

CG5939

CG5958

CG5969

CG5972

CG5973

CG5983

CG5988

CG5992

CG5996

CG6000

CG6008

CG6009

CG6012

CG6014

CG6015

CG6016

CG6020

CG6022

CG6025

CG6028

CG6030

CG6034

CG6038

CG6046

CG6056

CG6058

CG6074

CG6084

CG6090

CG6092

CG6094

CG6105

CG6115

CG6123

CG6126

CG6131

CG6141

CG6143

CG6147

CG6151

CG6153

CG6155

CG6164

CG6174

CG6180

CG6186

CG6195

CG6196

CG6206

CG6213

CG6216

CG6222

CG6224

CG6226

CG6236

CG6246

CG6249

CG6253

CG6258

CG6259

CG6272

CG6287

CG6289

CG6295

CG6296

CG6298

CG6299

CG6302

CG6310

CG6311

CG6318

CG6329

CG6340

CG6341

CG6342

CG6343

CG6353

CG6369

CG6393

CG6398

CG6403

CG6409

CG6410

CG6416

CG6421

CG6426

CG6443

CG6444

CG6446

CG6447

CG6450

CG6455

CG6457

CG6459

CG6463

CG6465

CG6467

CG6476

CG6483

CG6494

CG6510

CG6513

CG6514

CG6515

CG6523

CG6530

CG6533

CG6540

CG6543

CG6544

CG6554

CG6567

CG6579

CG6580

CG6583

CG6584

CG6585

CG6586

CG6593

CG6598

CG6610

CG6611

CG6620

CG6628

CG6638

CG6643

CG6644

CG6647

CG6666

CG6667

CG6672

CG6673

CG6692

CG6697

CG6704

CG6705

CG6712

CG6719

CG6746

CG6750

CG6755

CG6756

CG6757

CG6762

CG6764

CG6767

CG6770

CG6772

CG6773

CG6775

CG6776

CG6779

CG6801

CG6802

CG6803

CG6806

CG6808

CG6816

CG6818

CG6819

CG6821

CG6829

CG6838

CG6839

CG6840

CG6842

CG6846

CG6851

CG6852

CG6854

CG6859

CG6860

CG6869

CG6871

CG6875

CG6878

CG6879

CG6884

CG6891

CG6897

CG6912

CG6921

CG6933

CG6944

CG6948

CG6956

CG6959

CG6981

CG6982

CG6984

CG6988

CG6990

CG6998

CG7006

CG7007

CG7008

CG7009

CG7010

CG7012

CG7013

CG7016

CG7017

CG7023

CG7033

CG7037

CG7038

CG7046

CG7047

CG7048

CG7049

CG7054

CG7062

CG7066

CG7071

CG7072

CG7073

CG7077

CG7081

CG7107

CG7109

CG7111

CG7113

CG7118

CG7122

CG7123

CG7137

CG7160

CG7163

CG7168

CG7170

CG7171

CG7172

CG7175

CG7176

CG7178

CG7181

CG7188

CG7194

CG7196

CG7197

CG7199

CG7200

CG7206

CG7207

CG7224

CG7245

CG7246

CG7252

CG7257

CG7267

CG7269

CG7272

CG7281

CG7283

CG7287

CG7290

CG7291

CG7294

CG7298

CG7299

CG7301

CG7305

CG7319

CG7330

CG7338

CG7339

CG7343

CG7348

CG7354

CG7365

CG7375

CG7380

CG7382

CG7390

CG7392

CG7393

CG7394

CG7415

CG7421

CG7424

CG7425

CG7429

CG7430

CG7434

CG7435

CG7442

CG7462

CG7464

CG7478

CG7484

CG7490

CG7497

CG7498

CG7506

CG7508

CG7519

CG7523

CG7532

CG7539

CG7540

CG7550

CG7554

CG7567

CG7580

CG7581

CG7584

CG7590

CG7592

CG7593

CG7603

CG7610

CG7616

CG7619

CG7620

CG7622

CG7625

CG7630

CG7631

CG7636

CG7637

CG7639

CG7641

CG7646

CG7655

CG7658

CG7662

CG7663

CG7668

CG7669

CG7675

CG7678

CG7686

CG7694

CG7700

CG7710

CG7712

CG7713

CG7714

CG7715

CG7726

CG7738

CG7739

CG7748

CG7749

CG7752

CG7758

CG7762

CG7770

CG7772

CG7778

CG7787

CG7808

CG7820

CG7823

CG7830

CG7834

CG7842

CG7845

CG7866

CG7874

CG7876

CG7883

CG7885

CG7889

CG7891

CG7896

CG7911

CG7916

CG7917

CG7918

CG7920

CG7925

CG7930

CG7933

CG7936

CG7941

CG7943

CG7945

CG7946

CG7953

CG7954

CG7966

CG7968

CG7970

CG7971

CG7972

CG7973

CG7977

CG7981

CG7993

CG7997

CG7998

CG8003

CG8004

CG8009

CG8012

CG8013

CG8021

CG8024

CG8025

CG8026

CG8031

CG8038

CG8039

CG8044

CG8048

CG8050

CG8053

CG8055

CG8057

CG8065

CG8066

CG8068

CG8087

CG8090

CG8091

CG8097

CG8100

CG8108

CG8111

CG8117

CG8121

CG8128

CG8129

CG8132

CG8137

CG8144

CG8146

CG8149

CG8156

CG8160

CG8169

CG8180

CG8186

CG8189

CG8192

CG8194

CG8195

CG8203

CG8205

CG8206

CG8208

CG8209

CG8210

CG8226

CG8229

CG8230

CG8231

CG8238

CG8246

CG8251

CG8256

CG8261

CG8268

CG8272

CG8280

CG8297

CG8299

CG8302

CG8309

CG8310

CG8312

CG8314

CG8315

CG8320

CG8321

CG8322

CG8327

CG8328

CG8331

CG8332

CG8338

CG8340

CG8342

CG8353

CG8369

CG8372

CG8384

CG8385

CG8386

CG8392

CG8396

CG8402

CG8409

CG8411

CG8415

CG8416

CG8421

CG8430

CG8436

CG8439

CG8441

CG8444

CG8446

CG8451

CG8462

CG8472

CG8479

CG8481

CG8490

CG8493

CG8495

CG8498

CG8507

CG8510

CG8511

CG8515

CG8523

CG8526

CG8532

CG8542

CG8544

CG8549

CG8560

CG8577

CG8579

CG8580

CG8582

CG8583

CG8585

CG8588

CG8600

CG8604

CG8607

CG8610

CG8611

CG8614

CG8625

CG8627

CG8628

CG8629

CG8630

CG8636

CG8651

CG8660

CG8661

CG8669

CG8673

CG8674

CG8675

CG8676

CG8680

CG8697

CG8705

CG8715

CG8719

CG8722

CG8725

CG8733

CG8735

CG8743

CG8750

CG8759

CG8764

CG8781

CG8788

CG8790

CG8800

CG8804

CG8816

CG8818

CG8823

CG8827

CG8834

CG8839

CG8844

CG8846

CG8849

CG8857

CG8860

CG8861

CG8862

CG8863

CG8864

CG8866

CG8869

CG8871

CG8877

CG8882

CG8884

CG8885

CG8891

CG8892

CG8893

CG8900

CG8905

CG8908

CG8918

CG8920

CG8922

CG8927

CG8928

CG8936

CG8945

CG8946

CG8947

CG8952

CG8956

CG8963

CG8971

CG8972

CG8974

CG8978

CG8983

CG8986

CG8993

CG8994

CG8996

CG8997

CG8998

CG9000

CG9006

CG9008

CG9012

CG9020

CG9022

CG9027

CG9031

CG9032

CG9034

CG9035

CG9038

CG9040

CG9042

CG9056

CG9065

CG9066

CG9067

CG9073

CG9075

CG9077

CG9078

CG9080

CG9084

CG9088

CG9091

CG9095

CG9099

CG9107

CG9115

CG9120

CG9124

CG9126

CG9128

CG9140

CG9154

CG9159

CG9160

CG9172

CG9175

CG9181

CG9187

CG9193

CG9195

CG9205

CG9207

CG9212

CG9215

CG9231

CG9240

CG9244

CG9245

CG9250

CG9261

CG9267

CG9273

CG9277

CG9282

CG9286

CG9288

CG9291

CG9294

CG9295

CG9296

CG9298

CG9306

CG9319

CG9324

CG9325

CG9327

CG9331

CG9333

CG9334

CG9336

CG9338

CG9339

CG9344

CG9345

CG9350

CG9353

CG9355

CG9356

CG9358

CG9359

CG9362

CG9366

CG9372

CG9373

CG9375

CG9378

CG9383

CG9386

CG9388

CG9391

CG9396

CG9401

CG9410

CG9415

CG9422

CG9424

CG9427

CG9429

CG9434

CG9436

CG9450

CG9454

CG9456

CG9470

CG9471

CG9480

CG9488

CG9494

CG9496

CG9503

CG9504

CG9506

CG9525

CG9535

CG9536

CG9537

CG9538

CG9539

CG9540

CG9543

CG9548

CG9553

CG9556

CG9568

CG9578

CG9586

CG9588

CG9603

CG9614

CG9615

CG9617

CG9628

CG9631

CG9635

CG9638

CG9643

CG9648

CG9662

CG9667

CG9669

CG9670

CG9673

CG9674

CG9677

CG9684

CG9688

CG9691

CG9696

CG9710

CG9730

CG9734

CG9740

CG9741

CG9742

CG9745

CG9757

CG9762

CG9765

CG9769

CG9771

CG9773

CG9779

CG9790

CG9796

CG9812

CG9821

CG9829

CG9836

CG9847

CG9849

CG9852

CG9853

CG9856

CG9873

CG9875

CG9877

CG9878

CG9879

CG9881

CG9882

CG9888

CG9893

CG9894

CG9895

CG9911

CG9914

CG9916

CG9921

CG9922

CG9926

CG9928

CG9946

CG9948

CG9954

CG9968

CG9983

CG9990

CG9998

CG9999

CR30367

CR30425

CR31400

CR31511

CR31615

CR31696

CR31808

CR31931

CR31940

CR32314

CR32660

CR32665

CR32777

CR32863

CR32864

CR32886

CR32957

CR33258

CR33318

CR33328

CR40454

CR40456

CR40459

CR40474
